# Motion and emotion: depression reduces psychomotor performance and alters affective movements in caregiving interactions

**DOI:** 10.3389/fnbeh.2015.00026

**Published:** 2015-02-17

**Authors:** Katherine S. Young, Christine E. Parsons, Alan Stein, Morten L. Kringelbach

**Affiliations:** ^1^Department of Psychiatry, Warneford HospitalOxford, UK; ^2^MindLab/Center of Functionally Integrative Neuroscience (CFIN), Aarhus UniversityAarhus, Denmark; ^3^Department of Psychology, University of California, Los Angeles (UCLA)Los Angeles, USA

**Keywords:** depression, psychomotor performance, infant crying, social interaction, emotion

## Abstract

**Background**: Impaired social functioning is a well-established feature of depression. Evidence to date suggests that disrupted processing of emotional cues may constitute part of this impairment. Beyond processing of emotional cues, fluent social interactions require that people physically move in synchronized, contingent ways. Disruptions to physical movements are a diagnostic feature of depression (psychomotor disturbance) but have not previously been assessed in the context of social functioning. Here we investigated the impact of psychomotor disturbance in depression on physical responsive behavior in both an experimental and observational setting.

**Methods**: In Experiment 1, we examined motor disturbance in depression in response to salient emotional sounds, using a laboratory-based effortful motor task. In Experiment 2, we explored whether psychomotor disturbance was apparent in real-life social interactions. Using mother-infant interactions as a model affective social situation, we compared physical behaviors of mothers with and without postnatal depression (PND).

**Results**: We found impairments in precise, controlled psychomotor performance in adults with depression relative to healthy adults (Experiment 1). Despite this disruption, all adults showed enhanced performance following exposure to highly salient emotional cues (infant cries). Examining real-life interactions, we found differences in physical movements, namely reduced affective touching, in mothers with PND responding to their infants, compared to healthy mothers (Experiment 2).

**Conclusions**: Together, these findings suggest that psychomotor disturbance may be an important feature of depression that can impair social functioning. Future work investigating whether improvements in physical movement in depression could have a positive impact on social interactions would be of much interest.

## Introduction

Emotions are expressed in, and are also affected by, bodily actions and movements (Niedenthal, 2007). The notion of a reciprocal link between emotion and movement has intuitive appeal, but also considerable theoretical and empirical support. Indeed, the assertion that emotion must be understood in terms of bodily interactions with the world is a central tenet of embodied cognition approaches (Wilson, 2002). Empirical evidence shows that when an individual’s movement is disrupted, so too is their emotional experience (Niedenthal et al., 2005). Disrupted movement, or psychomotor disturbance, is a core diagnostic feature of many disorders of emotion (e.g., Bauer et al., 1991; Schrijvers et al., 2008), perhaps the most apparent of which is major depressive disorder.

While the key diagnostic criteria for depression are lowered mood and anhedonia, the disorder also entails significant abnormalities in psychomotor function (Whybrow and Mendels, 1969; for review, see Sobin and Sackeim, 1997; Canbeyli, 2010). Adults with depression demonstrate decreased overall motor activity (Wehr et al., 1980; Wolff et al., 1985), slower motor response times (Schwartz et al., 1989) and disrupted gross and fine motor movements relative to comparison groups (for review, see Schrijvers et al., 2008). However, despite evidence for its pervasiveness, psychomotor disturbance is one of the least understood of the core symptoms of depression.

Emotion processing in depression, by contrast, has been the subject of extensive investigation. Adults with depression show negative biases in information processing in a variety of domains (for reviews, see Clark et al., 2009; Harmer et al., 2009) including in response to lexical and social stimuli (e.g., Joormann and Gotlib, 2006; Bistricky et al., 2011). Within the social domain, there is evidence of disrupted processing of emotions in faces (Surguladze et al., 2005; Joormann and Gotlib, 2006; Stein et al., 2010; Arteche et al., 2011) and voices (e.g., Donovan et al., 1998; Schuetze and Zeskind, 2001; Péron et al., 2011; Young et al., 2012). Such disruptions have implications for an individual’s ability to navigate the social world and there is evidence to suggest that deficits in social functioning are an important factor in the maintenance of depression (Hammen, 1997; Joiner, 2002).

Psychomotor disturbance may also have consequences for social functioning in depression. Social interactions require the ability to detect emotional content and respond contingently (Kringelbach and Rolls, 2003). A critical feature of fluent interactions is synchronized physical movements, such as imitation (Heyes, 2011). Adults spontaneously mimic a variety of behaviors, including emotional facial expressions, manual gestures, body postures, mannerisms, and speech patterns (Chartrand and Bargh, 1999). Disorders involving disrupted psychomotor abilities, such as Parkinson’s disease, have been associated with disruptions to social functioning, including the ability to imitate facial expressions (Simons et al., 2003). How disruptions to emotional processing and psychomotor functioning in depression interact and potentially impact on social interactions is not currently understood.

## Parent-infant interactions as a model of affective social functioning

Parental caregiving inherently involves both reactions to emotional nonverbal cues and intricate, coordinated patterns of psychomotor activity. Parental care is frequently elicited by the sound of an infant cry, which typically evokes a strong emotional reaction in the listener (e.g., Frodi et al., 1978). Parent-infant interactions also tend to be highly synchronized, with both partners acting to maintain proximity to one another (Bowlby, 1969; Papousek and Papousek, 1989).

Previous work has shown that hearing a distressed infant’s cry can specifically improve adults’ ability to move in an effortful and coordinated manner (Parsons et al., 2012a). In addition, physiological reactivity to infant crying, indexed by increased heart rate, has been linked to sensitivity of maternal caregiving behavior (Del Vecchio et al., 2009; Joosen et al., 2013). Hearing infant vocalisations is also associated with early activity in the brainstem, a region that regulates autonomic function and responses to biologically salient information (Parsons et al., 2013b). Neuroimaging studies have also demonstrated increased activity in regions involved in arousal and emotion regulation (such as the amygdala and orbitofrontal cortex) when listening to infant cries (Lorberbaum et al., 1999; Swain et al., 2007; Bos et al., 2010; Kim et al., 2011). It has therefore been suggested that hearing an infant cry may induce a ‘high-alert’ state of autonomic arousal, where adults are physiologically primed to react to an infant’s distress (Parsons et al., 2012a, 2014b).

There is much interest in the role of gender in responsiveness to infant cues given the near universal evolutionary differences in the provision of caregiving between mothers and fathers. Studies in the visual domain (responding to images of infant faces) have demonstrated that women are more sensitive than men to infant “cuteness”, based on measures of explicit appraisal (“liking” ratings) and physiological reactivity (Sprengelmeyer et al., 2009; Parsons et al., 2011a; Esposito et al., 2014). However, measures of motivational salience (“wanting”) have found no differences between men and women in their ‘willingness to work’ to view images of infants (Parsons et al., 2011a; however, see also Hahn et al., 2013). In the auditory domain, women and men are similar in their explicit appraisal of infant vocalisations, reporting similar levels of perceived distress and desire to respond (Donate-Bartfield and Passman, 1985; Leger et al., 1996; Parsons et al., 2014b,c). There is mixed evidence on whether there are gender differences in physiological reactions to infant cries, with findings demonstrating greater reactivity in women than men (Wiesenfeld et al., 1981; Furedy et al., 1989) or greater reactivity in men than women (Brewster et al., 1998; Out et al., 2010). One study to date assessing motivational salience of infant cries demonstrated no gender differences (Parsons et al., 2012a).

## Evidence for disrupted social functioning in postnatal depression

Postnatal depression (PND) is defined as an episode of depression experienced by parents in the early months following childbirth. It has been identified as a global health issue (Parsons et al., 2012b; Howard et al., 2014; Stein et al., 2014) because it can compromise the quality of early care the child receives (Bigelow et al., 2010). Specifically, impairments in parental sensitivity to infant cues have been observed (e.g., Lester et al., 1995). PND has also been associated with a raised risk for childhood cognitive and socio-emotional problems (van Ijzendoorn et al., 1999; Murray et al., 2010). Mothers with PND have been shown to rate cries as less perceptually salient and less likely to elicit active caregiving responses than healthy mothers (Schuetze and Zeskind, 2001). Several studies have reported that mothers with depression are less likely to initiate appropriate caregiving responses to their infant’s cries than healthy mothers (Bettes, 1988; Murray et al., 1993; Schuetze and Zeskind, 2001). Disrupted sensitivity to distress in infant cries, as indexed by pitch, has also been demonstrated in both mothers with PND (Donovan et al., 1998) and adults with depression (Young et al., 2012). In addition, one recent study demonstrated that depressive symptoms were associated with lower than predicted physiological reactivity to infant crying (Riem et al., 2011). How depression impacts on motor aspects of parental sensitivity, such as the ability to move in reaction to the infant, has not been directly investigated.

## Study aims

In the current study, we employed two means of exploring the relationship between depression and psychomotor functioning. First, we assessed psychomotor performance in adults with and without depression using a standardized laboratory task of effortful, precise movement (Experiment 1). Second, we assessed physical movement in real-life emotive, social interactions between mothers with their infants (Experiment 2).

## Experiment 1

Experiment 1 aimed to investigate the impact of depression on two aspects of behavior important for social functioning: emotional processing and psychomotor performance. Responding to infant distress vocalisations requires rapid, co-ordinated movement, but also recognition of the emotional salience of the sound. We hypothesized that depression might impact on performance in either of two ways. One possibility is that differential responses to emotional cues are disrupted in depression. In this case, salient emotional stimuli would no longer hold a “privileged status” and would not promote faster coordinated effortful movements. A second possibility is that psychomotor disturbance in depression disrupts the ability to move quickly in response to all cues. This would result in overall slower responses in adults with depression, compared to healthy adults. Importantly, it would be expected that enhanced psychomotor performance after exposure to infant cries would still be present.

## Methods

### Participants

Table 1 presents participant demographic information. Twenty adults with current major depression (assessed using the Structured Clinical Interview for DSM-IV; SCID) and twenty adults without depression participated. This study was approved by the Oxfordshire Research Ethics Committee (12/07/2010). Participation was voluntary and all participants gave written informed consent.

**Table 1:** **Demographic characteristics of participants with and without depression**.

	Adults with depression	Adults without depression
*n* (*n* male)	20 (7)	20 (8)
Age in years *M (SD)*	27.55 (7.42)	28.50 (9.83)
EPDS score *M (SD)*	18.85 (2.56)*	3.25 (3.17)
GAD-Q score *M (SD)*	9.51 (1.21)*	1.94 (2.26)

*Denotes significant group differences (two-sample *t*-test, *p* < 0.001).

Participants were recruited from the student and general population through poster and online advertisements. Inclusion criteria were: no medication affecting the brain (including medications for the treatment of depression or anxiety) and no self-reported hearing impairments. Six participants (three healthy and three with depression) had children, all of whom were aged over 18 months at the time of testing. Participants were identified as experiencing moderate or severe depressive symptoms if they scored greater than or equal to 13 on the Edinburgh Postnatal Depression Scale (EPDS; a cut off with high sensitivity and specificity). The EPDS has been validated for use with women outside the postnatal period (Cox et al., 1996) and fathers (Matthey et al., 2001). It has also been used in a number of studies of men outside the postnatal period (e.g., Ramchandani et al., 2008).

Participants scoring above the threshold on the EPDS were then assessed using the SCID (using the following modules: mood episodes; anxiety disorders; obsessive-compulsive and related disorders; trauma and stressor-related disorders). Assessments were carried out by a trained psychologist and a second psychologist provided additional ratings of each interview. Given the high co-morbidity of depression and anxiety symptoms (approximately 35% in this sample), only participants who received a primary diagnosis of major depressive disorder were included in the study. Participants in this study reported high levels of depressive symptoms (EPDS scores; *M* = 18.85, *SD* = 2.56), well within the range for major depression (>13, Cox et al., 1996).

### Experimental task

Motor performance was assessed using the “Whack-a-mole” game, which requires participants to press randomly illuminating buttons, within a predetermined time and with a specific amount of force, in order to score points. Over the course of the game, the time limit for responses gets shorter as the lights are illuminated in quicker succession. The game therefore tests the accuracy of participants’ rapid, effortful responses. Participants were first familiarized with the game by playing three practice rounds of 30 s each.

Participants then played a full round of the “Whack-a-mole” game (lasting 1 min) after listening to 4.5 min of one of three types of sounds: infant cries, adult cries and bird sounds (for further details of the sound stimuli, see Parsons et al., 2012a; Young et al., 2012; Parsons et al., 2014b). This procedure was then repeated for each of the other two sound types, with the order counterbalanced across participants.

Performance was video recorded and subsequently coded. Total scores on the game were taken as a measure of accuracy. The force applied during the game was measured using a set of digital electronic scales (Salter 1036 BKDR, calibrated by the manufacturer) and coded to obtain a value for each individual button press. From this, mean, minimum and maximum pressure scores were extracted. Mean pressure was calculated as the average pressure applied during a single game and maximum and minimum pressure scores were extracted by taking the greatest and least amount of force applied, respectively. A factor analysis was performed to investigate the relationship between these dimensions (mean, maximum and minimum pressure) and whether variance might better be explained by an underlying latent variable, relating to “force” of motor responses. A 2 × 3 mixed ANOVA was used to analyze the accuracy and pressure data separately, with group as the between-subjects factor (depression, no depression) and stimulus category as the within-subjects factor (infant cry, adult cry, bird sound).

## Experiment 2

In Experiment 2, we assessed whether psychomotor impairments would be apparent during naturalistic social interactions. Using observations of real-life interactions of mothers and infants, we assessed the impact of PND on aspects of a mother’s physical movements towards her infant. We hypothesized that mothers with PND would show disruption to affective physical behaviors compared to mothers without PND.

## Methods

Data consisted of experimenter-coded observations of 54 mothers interacting with their infants, collected as part of a larger study of parent-infant interactions (the Oxford Parent Project, OPP). Data in the current study was taken from an experimental session conducted with mothers and infants at 10 months postpartum. This session included assessment of maternal mental health and observation of mother-infant interactions. Approval for the OPP study was obtained from the Oxfordshire Research Ethics Committee.

### Participants

Mothers were recruited to participate in a longitudinal observational cohort study (the Oxford Parent Project) from postnatal wards of the John Radcliffe Hospital, Oxford, UK. All mothers were aged 18 or over, spoke English, had no medical complications, were over 35 weeks gestation, and were the infants’ principal caregiver. All infants had a birth weight above 2000 g. Maternal mental health was assessed using the Structured Clinical Interview for DSM Axis I disorders (SCID, research version; First et al., 2002). The SCID was conducted by a trained researcher who also provided clinician’s severity ratings (CSR; Brown et al., 2001). The CSR is a scale ranging from 0–8, which indicates the level of distress or impairment associated with a specific symptom cluster (ratings of >4 indicate clinical severity).

Of the 253 participants who completed the 10-month assessment as part of the OPP, 30 were identified as fulfilling the criteria for current major depressive disorder. Of these 30 women, 27 had full ratings on all the observational dimensions being assessed in this experiment. Three were excluded because it was not possible to distinguish some of the psychomotor touch ratings assessed in this experiment. The group of 27 mothers included in this experiment had an average CSR of 5.15 (*SD* = 0.95). Participants were also assessed for other psychiatric disorders, demonstrating a substantial comorbidity of generalized anxiety disorder (GAD, 14 participants received CSR ratings >4 for GAD). The 27 participants with MDD were those who received a primary diagnosis of depression (CSR for GAD: *M* = 2.81, *SD* = 2.62). Of these participants, 13 reported that they were currently taking anti-depressant medication or receiving psychological treatment/counseling. A group of 27 mothers reporting no current psychiatric disorder (screened using the SCID) were selected to provide a control group against which to assess maternal behavior in PND. These participants were selected from the larger sample of OPP participants such that there were two groups of mothers matched as closely as possible: first for age (in years), then by socio-economic status and finally by parity (see Table 2).

**Table 2:** **Basic demographic details of participants**.

	Mothers with depression	Mothers without depression
Age in years *M (SD)*	33.19 (5.61)	33.19 (5.61)
Maternal SES *M (SD)*	3.80 (4.62)	5.19 (3.92)
% primiparous mothers	41%	67%
MDD CSR *M (SD)*	5.15, 0.95	–
GAD CSR *M (SD)*	2.81, 2.62	–

*Note. No significant differences in age, SES or parity were observed between groups (all *p* > 0.05)*.

### Interaction coding

Mother-infant interactions were recorded during 3.5 min free play sessions in which mothers were given a toy and instructed to play with their infant in any way they chose. Video tapes were subsequently coded on a number of dimensions relating to the quality of maternal responsiveness to the infant and the extent of maternal interactions. Ratings were completed by a trained researcher who was blind to the mother’s mental state (see Stein et al., 2012). For the current study, a subset of coded dimensions were selected to assess features of maternal psychomotor behavior and responses to emotional stimuli. Across the larger OPP study, a subsample of tapes was coded by a second trained researcher to assess inter-rater reliability of perceived maternal behaviors. Across 25 samples, inter-rater reliability on the dimensions included in this study was high (average weighted *K* = 0.72).

Coded dimensions relating to maternal psychomotor behavior consisted of “emotional touch”, “instrumental touch” and “strong control”. Emotional touch was coded as the “frequency of mother-infant contact for emotional reasons (warmth, caring, affection)”, on a 5-point Likert scale, ranging from 1, no touching, to 5, touching throughout most of the session. Instrumental touch was coded as “mother-infant contact for non-emotive, mechanical reasons”, using the same 5-point Likert scale as for emotional touch. Strong control was coded as “the extent to which mother uses greater power or strength to override the child”, on a 3-point Likert scale, ranging from 1, no strong control, to 3, consistently uses strong control.

Coded dimensions relating to maternal responses to infant cues consisted of general “maternal withdrawal” and specific “responses to vocal cues”. Maternal withdrawal was coded as “mother’s lack of engagement/interaction with her infant” on a 5-point Likert scale ranging from 1, very withdrawn, to 5 not at all withdrawn. Responses to vocal cues were coded as “the extent to which the mother picks up on the child’s vocal cues, recognizing his/her vocalisations in an appropriate way”, on a 5-point Likert scale, ranging from 1, very poor (little response to vocalisations), to 5, very good (reacting to almost all vocalisations, repeating and expanding). Ordinal data from Likert scales of coded maternal behavior were compared between mothers with and without PND using non-parametric statistical tests (independent-samples Mann-Whitney *U* tests).

## Experiment 1: results

Factor analysis demonstrated that the three force measure variables were significantly positively correlated. Mean pressure scores were highly correlated with maximum pressure scores (*r* = 0.91) and minimum pressure scores (*r* = 0.46). Maximum and minimum pressure scores were also strongly correlated (*r* = 0.39). A principal components factor analysis on standardized, log-transformed scores demonstrated that all three pressure measures loaded onto one underlying “pressure factor”, explaining 73.69% of the variance in the data, with an eigenvalue of 2.21. This factor reflected overall effort during the game, while also reflecting peak performance (as indexed by maximum scores) and sustained effort (minimum scores).

**Figure 1:**
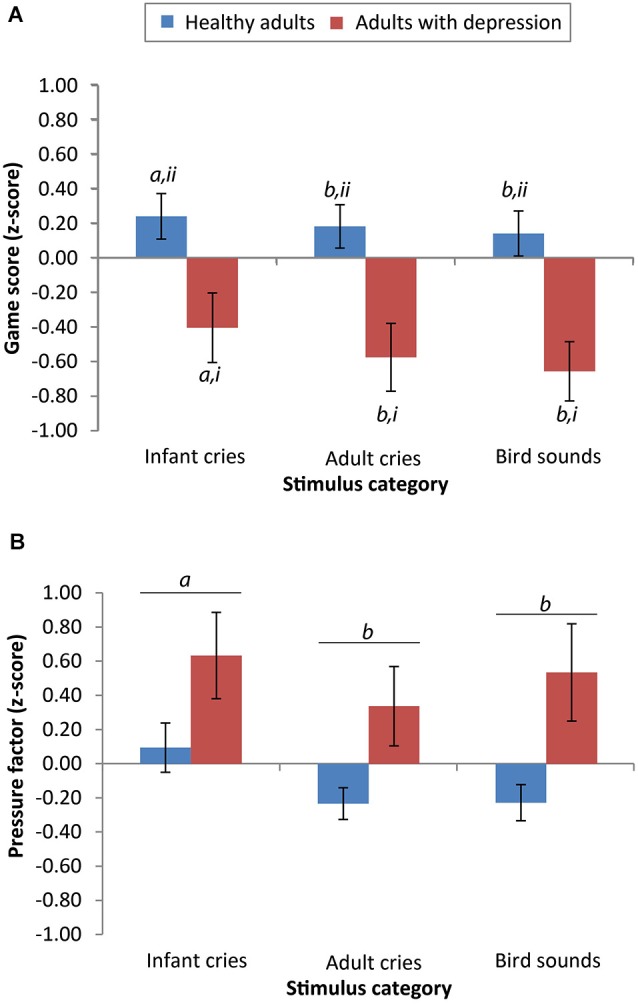
**Performance of adults with depression is significantly lower on the effortful motor task than healthy adults, but differential reactivity to salient emotional cues remains evident in both groups. (A)** Scores from both groups were significantly higher after listening to infant cries compared to listening to adult cries or bird sounds (means with different letters are significantly different, *a* > *b, p* < 0.05). The mean scores of healthy adults on the “Whack-a-mole” game were significantly higher than the scores of adults with depression (means with different numerals are significantly different, *ii* > *i*, *p* < 0.05). **(B)** In both groups, pressure applied was greater after listening to infant cries compared with adult cries or bird sounds (means with different letters are significantly different, *a* > *b*, *p* < 0.05). Error bars reflect mean +/− standard error.

For the accuracy data, there was a significant main effect of group (*F*_(1,38)_ = 7.37, *p* = 0.01, *r* = 0.40) and sound type (*F*_(2,76)_ = 6.66, *p* = 0.002, *r* = 0.28), but no significant interaction between the two (*F*_(2,76)_ = 1.23, *p* = 0.30, *r* = 0.13). Across all sound categories, scores from adults with depression (*M* = 35.60, *SD* = 22.05) were significantly lower than scores from the healthy adults (*M* = 55.62,* SD* = 24.52). *Post hoc* least squares difference (LSD) comparisons showed that across both participant groups, scores on the game were significantly higher after listening to infant cries (*M* = 48.63, *SD* = 26.68) compared to adult cries (*M* = 44.25, *SD* = 25.94; *p* = 0.005) and compared to bird sounds (*M* = 43.95, *SD* = 24.40; *p* = 0.002). There was no significant difference in game scores after listening to adult cries compared to bird sounds (*p* = 0.84; see Figure 1).

For the pressure data, taking the “force” variable, there was no significant main effect of group (*F*_(1,34)_ = 0.71, *p* = 0.41, *r* = 0.14) but there was a significant main effect of stimulus category on pressure factor scores (*F*_(2,68)_ = 3.98, *p* = 0.02, *r* = 0.24). There was no significant interaction between group and stimulus category (*F*_(2,68)_ = 0.65, *p* = 0.53, *r* = 0.10). Participants with depression did not differ in the amount of applied pressure (pressure factor scores: *M* = 0.25, *SD* = 0.95) compared with healthy participants (*M* = −0.01, *SD* = 1.04). *Post hoc* LSD comparisons demonstrated that across both participant groups, pressure factor scores were significantly higher after listening to infant cries (*M* = 0.31, *SD* = 1.03) compared to adult cries (*M* = 0.09, *SD* = 1.00; *p* < 0.001) and compared to bird sounds (*M* = 0.07, *SD* = 1.00; *p* = 0.04). There was no significant difference in pressure applied after listening to adult cries compared to bird sounds (*p* = 0.67; see Figure 1).

There were no significant effects of gender on task performance as demonstrated by 2 × 2 × 3 mixed ANOVAs, with gender and participant group as between-subject factors and stimulus category as a within-subject factor. For the game score data, there was no significant main effect of gender (*F*_(1,36)_ = 0.13, *p* = 0.72), no significant interaction of gender with participant group (*F*_(1,36)_ = 0.35, *p* = 0.56) and no significant interaction of gender with stimulus category (*F*_(2,72)_ = 0.1.98, *p* = 0.15). Similarly, for the pressure factor data, there was no significant main effect of gender (*F*_(1,32)_ = 1.76, *p* = 0.19), no significant interaction of gender with participant group (*F*_(1,32)_ = 0.58, *p* = 0.45) and no significant interaction of gender with stimulus category (*F*_(2,64)_ = 1.22, *p* = 0.30).

## Results: experiment 2

**Figure 2:**
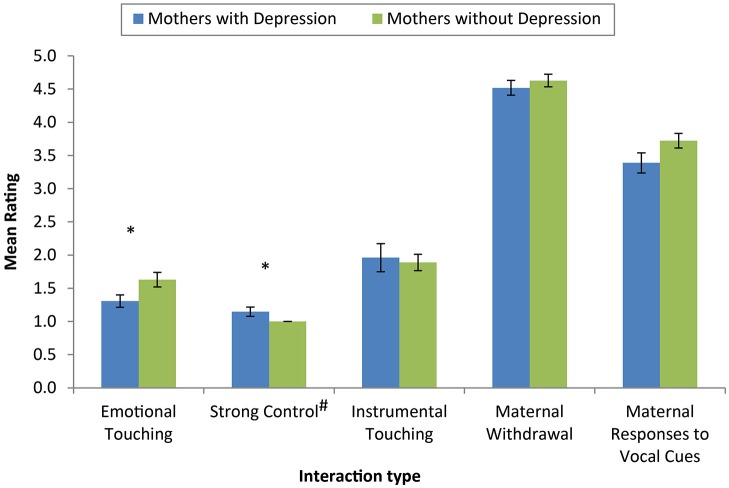
**Mean ratings of dimensions of coded interactions across mothers with and without depression**. Mothers with depression showed less emotional touching and more strong control than mothers without depression. There were no significant differences between groups on instrumental touching, maternal withdrawal or maternal responses to infant vocal cues. (* Denotes significantly different values, *p* < 0.05, error bars reflect mean +/− standard error, # note that strong control was coded on a 3 point scale).

Significant differences in the extent of both emotional touching (*U* = 247.00, *p* = 0.03, *r* = −0.32) and the use of strong control (*U* = 418.50, *p* = 0.04, *r* = 0.28) were observed (see Figure 2). While the size of the effects were small to medium, mothers with PND demonstrated less emotional touching and more “strong control” compared with mothers without PND. There was no significant difference in the extent of instrumental touching (*U* = 330.05, *p* = 0.69, *r* = −0.06) between mothers with and without PND. No significant differences were observed between mothers with and without PND in coded measures of maternal withdrawal (*U* = 332.50, *p* = 0.52, *r* = −0.09) or maternal responses to infant vocal cues (*U* = 122.00, *p* = 0.16, *r* = −0.23).

## Discussion

These findings suggest that depression is associated with disruptions to psychomotor abilities, which could play a role in social functioning. This is supported by two converging lines of evidence. First, we found that adults with depression demonstrated reduced effortful motor performance compared with healthy adults. Despite this disruption, the performance of both groups of adults was enhanced after listening to sounds of greatest emotional salience. This highlights the persistent nature of psychomotor disruption in depression, which impacts on behavior even when reactivity to highly emotive social cues is retained. Second, we found that mothers with PND demonstrated altered physical movements during interactions with their infants compared with healthy mothers.

In Experiment 1, after listening to infant cries, participants’ motor performance was more accurate and more forceful than after listening to adult cries or bird sounds. This suggests that hearing the highly salient sound of a distressed infant has a similarly rousing effect in adults with and without depression. Despite this reactivity to infant cries, the performance of adults with depression was impaired relative to that of healthy adults. These findings are consistent with the view that depression may have a general impact on psychomotor abilities (for review, see Schrijvers et al., 2008). In addition, there were no observed gender differences in the motivational salience of infant cries, in line with previous research (Parsons et al., 2012a).

The overall difference between the accuracy scores of the adults with and without depression was substantial. On average, healthy adults had scores that were 66% higher than adults with depression. This indicates a pervasive disruption to motor performance in adults with depression on this task. Of note, adults with depression did not differ from healthy adults in the amount of pressure applied while playing the game. This suggests that the lower overall scores of the adults with depression were a consequence of slower and less accurate movements, rather than reduced force of individual movements. While general psychomotor disturbance in depression has been widely reported (Moffoot et al., 1994; Buyukdura et al., 2011), our findings suggest two dissociable components: speed and accuracy of a movement, and the amount of force applied. Our results indicate that while the former is disrupted in depression, both components are enhanced after hearing infant cries.

The lack of enhanced motor performance after listening to adult cries suggests that while both adult and infant cries are important classes of emotional stimuli, there is something unique about the processing of infant cries. We suggest that the difference is in the communication of immediate need in infant crying, but not in adult crying. Adult crying can convey joy or sadness, depending on the context of the expression. Interpretation of genuine distress may also require other information, such as the visual cue of “tearing” (Provine et al., 2009). Our results suggest that infant cries, a class of urgent, emotional sounds, can elicit a sustained state of increased reactivity in adults with and without depression.

A physiological state that allows individuals to move with greater speed and accuracy upon hearing a distressed infant may be an adaptive mechanism that facilitates caregiving behavior. Neuroimaging studies of pain and aversion suggest that these types of mechanisms recruit affective brain areas (Lindquist and Barrett, 2012), disrupted activity in which has long been a core feature of brain models of depression (e.g., Mayberg, 1997). Studies of neural responses to infant communicative cues suggest similar networks are recruited as part of the “parental brain” (Swain et al., 2008; Bos et al., 2010; Parsons et al., 2010; Kim et al., 2011; Laurent and Ablow, 2012). One region in particular, the orbitofrontal cortex, is thought to be critically involved in the rapid processing of infant cues (Kringelbach et al., 2008; Parsons et al., 2013c). A breadth of behavioral evidence now supports the notion of privileged processing of infant cues (Sprengelmeyer et al., 2009; Parsons et al., 2011a,b, 2013a). In addition, recent evidence suggests that physical aspects of parent-infant interactions might be specifically linked to functioning of the oxytocinergic system (Weisman et al., 2013). Future studies investigating cortical and neuroendocrine processes involved in adults’ behavioral responses to infant cues would further inform this emerging field.

We hypothesized that maternal PND would be associated with altered physical movements during mother-infant interactions. Previous work has demonstrated that parent-infant interactions are often disrupted in PND (e.g., Stein et al., 1991; Murray et al., 1996). In the current study, mothers with PND showed differences in key aspects of affective, physical movements during interactions with their infants compared with mothers without PND (Experiment 2). These small, but significant, differences were apparent in mothers’ use of “emotional touch” and “strong control”, but not in non-affective “instrumental touch”, or other aspects of maternal responsiveness. Our findings, while preliminary, suggest important differences in psychomotor capacities related specifically to affective physical interactions between mother and infant.

The majority of previous work examining maternal responses to infant cries in PND has been experimental in nature (e.g., Lester et al., 1995; Donovan et al., 1998). This previous work has primarily focused on assessing specific responses to subtle differences in infant cry acoustics in experimental settings. In contrast, Experiment 2 used a naturalistic observational setting with spontaneous, idiosyncratic infant vocalisations. We found no significant disruption in maternal responses to infant cues as a category of stimuli in depression. This suggests that there may not be an obvious overall impairment in responding to infant vocalizations, at least as measured by a 5 point Likert scale. Instead, depression may impact the ability to detect and interpret subtle differences in vocal characteristics.

The specificity of differences in maternal behavior to affective aspects of physical touch is of particular interest. There is mounting neuroscientific evidence for a dissociation between “discriminative” and “affective” aspects of touch (for review, see McGlone et al., 2014). It has been demonstrated that infants are sensitive to this distinction, showing more signs of reward (i.e., smiling) to affective stroking, than to passive touch (Stack and Muir, 1990, 1992; Jean et al., 2009). Our findings suggest that depression may disrupt affective physical interactions between mother and infant. However, it is unclear at this stage whether this linked to changes in psychomotor behavior, affective processing, or some combination of the two.

Interventions targeting affective physical behavior have previously shown positive effects for infant development. Close physical skin-to-skin contact between mother and infant has been shown to confer benefits for child development in cognitive and motor outcomes (for review, see Tessier et al., 1998). Increasing skin-to-skin contact has also been shown to reduce depressive symptoms in mothers (e.g., Tessier et al., 1998; Feldman et al., 2002). The present finding of observable changes in mothers’ physical movements during interactions with their infants in PND provides further impetus for exploring affective physical behavior as a target for intervention.

### Strengths and limitations

A key strength of this study is that we present evidence for functionally salient motor impairments in depression using independent experimental and observational methods, testing different samples of participants. The results from these two methods are also convergent. We demonstrate that psychomotor disturbance, apparent in current and previous laboratory based tests of motor function (e.g., Schwartz et al., 1989; Sobin and Sackeim, 1997; Schrijvers et al., 2008), can also be measured in social interaction behaviors. However, our findings are exploratory in nature, especially given that the recorded social interaction consisted of brief (3.5 min), laboratory-based mother-infant interactions. Furthermore, effect sizes were small, which is perhaps related to the three to five point scales on which touch behaviors were coded, limiting the sensitivity of this measure to subtle differences in behavior. In addition, while we assessed participants for disorders related to emotional processing, we did not specifically assess changes in other capacities (such as attention) that may have an impact on performance in the measures used. Future studies might examine individual differences in other factors related to caregiving such as empathy, parity and infant temperament (Decety and Cowell, 2014; Parsons et al., 2014a). Finally, it remains to be seen whether psychomotor disruption in depression has a similar impact on physical movements during interactions between adults.

## Conclusion

Psychomotor disturbances and their impact on social interaction should be considered alongside other well-established deficits in social cue processing (e.g., Joormann and Gotlib, 2006; Stein et al., 2010; Arteche et al., 2011; Naranjo et al., 2011). However, our findings suggest that the role of psychomotor disturbances may have been underestimated, or at least underspecified, in prior conceptualizations of social functioning in depression. Current models of embodied emotion, which specify an intricate link between emotion and movement, lend theoretical credence to this notion. Altered movement patterns were apparent in adults with depression both in an experimental task requiring precise, co-ordinated movements, and in more naturalistic social interactions. Disrupted emotional experience, which characterizes depression, may be associated with changes in the ability to move in functionally important ways.

## Financial support

This research was supported by a Medical Research Council Studentship to Katherine S. Young, funding from the Welcome Trust, United Kingdom (Grant071571), and the TrygFonden Charitable Foundation and ERC Consolidator Grant CAREGIVING (n.615539).

## Conflict of interest statement

The authors declare that the research was conducted in the absence of any commercial or financial relationships that could be construed as a potential conflict of interest.
